# Canonical correlations between individual self-efficacy/organizational bottom-up approach and perceived barriers to reporting medication errors: a multicenter study

**DOI:** 10.1186/s12913-019-4194-y

**Published:** 2019-07-16

**Authors:** Myoung Soo Kim, Chul-Hoon Kim

**Affiliations:** 1 Department of Nursing, Pukyong National University, 599-1, Daeyeon 3 dong, Namgu, Busan, 48513 South Korea; 2 College of Medicine, Dong-A University Hospital, 26 Daesingongwon-ro, Seo-gu, Busan, 49201 South Korea

**Keywords:** Clinical decision-making, Intersectoral collaboration, Knowledge, Medication errors, Social perception

## Abstract

**Background:**

Individual and organizational factors correlate with perceived barriers to error reporting. Understanding medication administration errors (MAEs) reduces confusion about error definitions, raises perceptions of MAEs, and allows healthcare providers to report perceived and identified errors more frequently. Therefore, an emphasis must be placed on medication competence, including medication administration knowledge and decision-making. It can be helpful to utilize an organizational approach, such as collaboration between nurses and physicians, but this type of approach is difficult to establish and maintain because patient-safety culture starts at the highest levels of the healthcare organization. This study aimed to examine the canonical correlations of an individual self-efficacy/bottom-up organizational approach variable set with perceived barriers to reporting MAEs among nurses.

**Methods:**

We surveyed 218 staff nurses in Korea. The measurement tools included a questionnaire on knowledge of high-alert medication, nursing decision-making, nurse-physician collaboration satisfaction, and barriers to reporting MAEs. Descriptive statistics, t-tests, analysis of variance (ANOVA), Pearson’s correlation coefficient, and canonical correlations were used to analyze results.

**Results:**

Two canonical variables were significant. The first variate indicated that less knowledge about medication administration (− 0.83) and a higher perception of nurse-physician collaboration (0.42) were related to higher disagreement over medication error (0.64). The second variate showed that intuitive clinical decision-making (− 0.57) and a higher perception of nurse-physician collaboration (0.84) were related to lower perceived barriers to reporting MAEs.

**Conclusions:**

Enhancing positive collaboration among healthcare professionals and promoting analytic decision-making supported by sufficient knowledge could facilitate MAE reporting by nurses. In the clinical phase, providing medication administration education and improving collaboration may reduce disagreement about the occurrence of errors and facilitate MAE reporting. In the policy phase, developing an evidence-based reporting system that informs analytic decision-making may reduce the perceived barriers to MAE reporting.

## Background

Patient-safety reporting systems have been used to detect the root causes of errors and to enhance patient safety and the economic efficiency of healthcare [1]. However, the number of errors reported by these means is less than the number of actual errors determined by experts [2]. Because under-reporting errors is a critical limitation to analyzing the process behind medication administration errors (MAEs) [3], understanding perceived barriers to error reporting is crucial in promoting reporting behavior. Several studies have shown individual and organizational factors for enhancing error- reporting [2, 3], and one study [4] proposed a conceptual model for determinants of MAE reporting with an individual perceived self-efficacy and organizational factors such as reporting process capability and support, organizational culture, management support, and regulatory authority.

Individual perceived self-efficacy is defined as an individual’s ability or competence to effectively cope with responding to MAEs [4]. Understanding MAEs reduces confusion about error definitions [5], raises levels of clinical reasoning about whether the error was harmful [6], and allows more frequent reporting of perceived and identified errors by healthcare providers [5]. Therefore, required competencies for medication safety were a knowledge and decision-making [7]. Collection of audit data and reporting of errors provide evidence-based knowledge of medication administration standards [8]. However, the information accumulated to measure knowledge is insufficient, owing to the lack of feedback on error-reporting systems [8]. Furthermore, while the majority of MAEs do not result in harm [9], some drugs, known as ‘high-alert’ drugs, are more likely to cause significant patient harm when used in error [10]. More attention should be paid to substances categorized as high-alert drugs by the US Institute for Safe Medication Practices [10] and a systematic review [11], including anticoagulants, cardiovascular drugs, insulins, and opiates, among others. Specific knowledge about high-alert medication, which is high -risk medication for nurses’ MAEs [12], can serve as the basic competence level when judging what is an MAE and deciding whether to report it.

Judging medication errors and the decision to report them may depend on nurses’ knowledge base and their decision-making ability; however, there is insufficient previous research on nurses’ decisions to report errors. In particular, barriers to reporting MAEs may depend on whether decision-making is analytic or intuitive [13]. According to analytical-intuitive decision-making theory [14], cognitive processes may move along the analytical-intuitive continuum over time. Generally, analytical decision-making is known as an appropriate decision-making style when information is organized in memory and the correct weight and importance is given to each piece of information [14, 15]. Intuitive decision-making is defined as an unconscious, not logically defensible, and non-stepwise process [14, 15]. However, decision-making cannot be the same across diverse nursing situations [15], and little is known about the relationship between nurses’ decision-making and error- reporting situations.

Regarding organizational factors, improving patient-safety culture and supportive leadership is a key organization-based strategy for enhancing error reporting [4, 16]. Safety culture has major effects on patient outcomes and a positive safety culture in healthcare is evidenced by the visibility of leaders and support for patient-safety initiatives [17]. However, because patient-safety culture starts at the highest levels of the healthcare organization [16], it is difficult to establish and maintain supportive management of medication safety [18]. Therefore, it can be helpful to utilize a bottom-up approach [19]. A bottom-up approach to improving patient-safety culture means that patient-safety awareness should be embedded at all staff levels and be seen in all actions [20], and collaboration among healthcare providers can establish effective communication [21] and create a positive patient-safety culture. Communication openness regarding mistakes and failures is a crucial factor in patient-safety; however, communication remains an adaptive challenge that has proven difficult to overcome in the sociotechnical viewpoint that defines healthcare [22]. Most of the current literature has focused on a top-down approach such as supportive leadership for enhancing error reporting [16–18] and there has been limited research on bottom-up approaches such as collaboration.

Although several studies have demonstrated perceived barriers to error reporting, there is a gap in the empirical study of areas such as knowledge for errors, decision-making, and the organizational bottom-up approach. Furthermore, there may be combination effects of variables in relation to individual or organizational factors for perceived barriers to MAE reporting. Based on the fact that communication and pharmacological knowledge impact nurses’ decision-making concerning medication [23], research variables are expected to show something other than a bivariate relationship with perceived barriers to MAE reporting. Therefore, the current research questions were set as follows: (1) is there a relationship between an individual self-efficacy/organizational bottom-up approach set and perceived barriers to MAE reporting? And if so, (2) what factor has the strongest relationship for reducing perceived barriers to reporting MAE?

## Methods

### Study design and participants

This study employed a cross-sectional descriptive design and non-probability sampling was used. The address book of the Korean Nurses Association was used to create a list of hospitals. We were able to obtain the telephone numbers and e-mail addresses of the nursing administration departments at each hospital. Two hundred and seventy four secondary and tertiary hospitals were invited to take part, of which 28 responded to the first e-mail. Finally, the department heads of seven hospitals gave us permission to distribute the questionnaire after reviewing the research protocol and the IRB’s approval. The inclusion criteria for participating registered nurses (RNs) were a) having at least 3 months’ clinical experience (because the first 3 months are generally a job-related orientation period in Korea) and b) not administrative nurses, such as unit managers or charge nurses, because they are more likely than junior staff nurses to know how or what to do about reporting [24], potentially biasing the results.

To control possible confounding variables of work experience, job location, and job position, we asked the nursing department to distribute anonymous self-administered questionnaires to nurses in a minimum of three units in each hospital. Three to five units in each hospital were included in the data collection. A variety of wards (medical, surgical, rehabilitation, gynecology, pediatric) and intensive care units (medical, surgical, cardiac, neuroscience, traumatic) were included. Many hospitals limited the number of participants in order to minimize nurses’ additional workload. Therefore, 20 questionnaires were provided for one hospital with less than 500 beds, 30 questionnaires for two hospitals with 500–699 beds, 45 questionnaires for two hospitals with 700–999 beds, and 50 questionnaires for two hospitals with more than 1000 beds. We hired one nurse as a research assistant in each hospital to distribute and collect the data. They distributed questionnaires to staff nurses at the direction of the nursing department. Respondents placed completed, sealed questionnaires in a box located at the nurse station on each floor and research assistants collected the questionnaires.

A total of 270 staff nurses were surveyed from November 2012 to December 2013. The mean duration of questionnaire distribution and collection was 2 months. However, two hospitals gave late permission for survey research on August 2013, so the duration of questionnaire distribution and collection was 3 months, from September to December 2013. Two -hundred thirty-one questionnaires were returned (response rate of 85.6%). After excluding thirteen questionnaires whose respondents had less than 3 months of work experience (7) or had missing answers (6), 218 questionnaires were included in the analysis. The sample size was determined based on the recommendation that about ten cases are needed for every variable in the canonical correlation [25]. In this study, there are seven variables in the final analysis, so 218 participants would be acceptable.

### Outcome measures

#### Individual self-efficacy/organizational bottom-up approach set

##### Individual self-efficacy; knowledge of medication administration

High-alert medications may cause significant patient harm when used in error; however, high-alert drugs have not been categorized in Korea. Therefore, potassium chloride, insulin, morphine, heparin, and warfarin drugs were regarded as high-alert drugs based on a specific research [10]. To examine knowledge of medication administration, we used a 20-item questionnaire about “knowledge of high-alert medication” developed by Hsiao et al. [12] and verified in Korean by Kim and Jung [26]. The questionnaire had two parts: Part A evaluated drug administration with a focus on drug delivery routes and dosage; and Part B evaluated drug regulations with a focus on how high-alert medications should be stored, regulated, and documented. Therefore, knowledge of high-alert medication administration is considered to contain valuable information about medication errors. Five-points were given for a correct answer, resulting in a total possible score range of 0–100. The KR-20 value, an assessment of reliability, estimates the consistency of measurement using the variance of item scores and is used when the items are dichotomous. It can also estimate consistent reliability compared to the split-half estimation. Internal consistency in the previously reported KR-20 [12] was 0.74; in a previous study [26] it was 0.61, and in this study, it was .62, indicating relatively low reliability. However, a tentative social psychological instrument can be used for research if the reliability of the instrument is over .60 [27].

##### Individual self-efficacy; nursing decision-making

A 24-item nursing decision-making instrument was used, developed by Lauri and Salanterä [15] and verified in Korean by Kim and Jung [28]. This instrument was developed with reference to evidence that nurses flexibly apply diverse kinds of decision-making models depending on the specific nursing problem, the task at hand, the information available, organization level, and time constraints [15]. The instrument consisted of 24 items scored from one (definitely disagree) to five (definitely agree). Scores for responses to odd items were reversed, and those for even items were not changed [15]. Scores were summed; a low score indicated an analytic approach to decision-making, a high score an intuitive approach [15]. Cronbach’s alpha for this study was 0.87.

##### An organizational bottom-up approach; nurse -physician collaboration satisfaction

A nurse -physician collaboration satisfaction questionnaire [29] was used to investigate an organizational bottom-up approach. To assess semantic equivalence, translation and back-translation using a synthesis of translations by two bilingual professionals were conducted. Content validity was checked by two experts, one nursing professor and one nursing unit manager with a doctoral degree. The content validity index for relevance and representativeness was calculated for each item using a 4-point scale (4 = highly relevant, 3 = quite relevant, 2 = somewhat relevant, 1 = not relevant). I-CVI (item CVI) is 1.00, which is acceptable because Lynn’s recommendation [30] is that I-CVI should be 1.00 when there are five or fewer experts. Based on the Polit’s study [31], S-CVI (scale CVI) was valid because it is over 0.9. This instrument consisted of eight items with seven response options; six of the items ranged from “strongly disagree” to “strongly agree,” one ranged from “no collaboration” to “complete collaboration,” and one ranged from “not satisfied” to “very satisfied.” Higher scores indicated greater collaboration satisfaction between nurses and physicians in an organization. Cronbach’s alpha was 0.93 for this study.

#### Perceived barriers to reporting MAEs

The Barriers to Reporting MAEs instrument, which was developed by Wakefield, Wakefield, Uden-Holman and Blegen [32] and verified for semantic equivalence and content validity in Korean by the research team, was used. Two of the researchers translated the instrument into Korean using reverse-translation and bilingual techniques as suggested by Brislin [33]. I-CVI was 0.87 and S-CVI was 0.82 because the study included six experts who worked for the patient-safety departments of two hospitals. An exploratory factor analysis with varimax rotation was performed and items with component loadings greater than 0.3 were retained [25]. Factorability was examined using a Kaiser-Meyer-Olkin (KMO) test. Kaiser’s measure of sampling adequacy requires a value of 0.6 or above [34]; our test gave a KMO of .91, indicating good factor analysis. Three dimensions indicated a poor fit of the model to our data; we therefore carried out analysis to determine a four-dimension structure. As a result, total percentage of variance was 61.8% and the 16-item instrument included four dimensions (fear, disagreement over whether an error occurred, effort required to report MAEs, and administrative responses to medication errors). Higher scores corresponded to a higher perception of barriers to reporting MAE. Item responses were measured on a five-point agreement scale from one (definitely disagree) to five (definitely agree). Cronbach’s alpha for this study was 0.76, indicating that this instrument was suitable for a social psychological study.

### Ethical considerations

This study was conducted after being reviewed and approved by the institutional review board (IRB) at one of the participant hospitals in this study (IRB no. E-20120821). Several hospitals were unable to review and approve the study because they either did not have individual IRBs or did not have employees on the research team. Therefore, the research team recruited hospitals that gave their permission after reviewing the research protocol and the approval of one IRB. On the first page of the survey, the purpose, voluntary participation, confidentiality of the information, and procedures of the study were explained. Written consent was obtained from participants prior to questionnaire distribution.

### Data analysis

Data were analyzed using SPSS 23.0 (SPSS, Inc., Chicago, IL. USA). Descriptive analyses, independent t-tests, and ANOVA were used. Pearson correlation was used for screening assumption; the results of the correlation coefficient values were not interpreted. Canonical correlation analysis was employed after the assumptions of the canonical correlation were checked. Interpretation of reliable pairs of canonical variates was based on the loading matrices. Because the loading matrices contained correlations, and squared correlations measure overlapping variances [25], variables with correlations of 0.30 and above were interpreted.

Internal consistency is usually calculated from the pairwise correlations between items and some social psychological instruments can have lower reliability [27]. Even though a commonly accepted rule of thumb for describing internal consistency is to deem it acceptable when it is between 0.7 and 0.8, an internal consistency over 0.6 is acceptable in this study because a tentative social science instrument was included [27].

## Results

### Perceived barriers to reporting MAE according to participants’ characteristics

The mean age of the participants was 28.6 years, and almost all participants (97.2%) were female. Only 1.3% of the participants had a master’s degree, while 76.6% were married. Twenty-eight percent of the participants had above 1 year and less than 3years of total nursing experience. There were no significant differences in perceived barriers to reporting MAEs; however, we observed a lower average rating for perceived barriers to reporting MAEs among participants with a master’s degree (F = 4.13, *p* = .017) (Table 1).

**Table 1:** Perceived Barriers to Reporting Medication Administration Errors according to Socio-demographic Characteristics of Participants

Characteristics		*n*(%)		(*N* = 218)
			Perceived barriers to reporting medication administration error	
			M ± SD	t or F(p)
Age	22–29	161(65.2)	2.71 ± 0.42	2.65(.073)
(28.60 ± 6.15)	30–39	48(25.9)	2.56 ± 0.38	
	Above 40	9(8.9)	2.72 ± 0.55	
Gender	Male	6(2.8)	2.38 ± 0.62	−1.82(.070)
	Female	212(97.2)	2.69 ± 0.41	
Education level	College	93(42.7)	2.64 ± 0.41	4.13(.017)
	University	122(56.0)	2.72 ± 0.41	
	Master’s degree	3(1.3)	2.08 ± 0.67	
Marital status	Single	51(23.4)	2.61 ± 0.46	−1.39(.165)
	Married	167(76.6)	2.70 ± 0.40	
Total experience in nursing (year)	< 1	37(17.0)	2.66 ± 0.43	0.17(.975)
	1- < 3	61(28.0)	2.67 ± 0.42	
	3- < 5	48(22.0)	2.72 ± 0.41	
	5- < 10	45(20.6)	2.67 ± 0.41	
	10- < 15	18(8.3)	2.64 ± 0.40	
	≥ 15	9(4.1)	2.71 ± 0.56	
Number of bed	< 500	13(6.0)	2.70 ± 0.50	0.31(.818)
	500- < 700	34(15.6)	2.66 ± 0.37	
	700- < 1000	83(38.1)	2.65 ± 0.44	
	≥1000	88(40.4)	2.71 ± 0.40	
Total mean score			2.66 ± 0.41	

### Knowledge about medication administration, nursing decision-making, nurse-physician collaboration satisfaction, and perceived barriers to reporting MAE

The correct-answer rate for knowledge of medication administration was 65%. The nursing decision-making mean score was 2.93 ± 0.11. The overall mean score of nurse-physician collaboration satisfaction was 4.01 ± 0.97. The mean score of perceived barriers to reporting MAEs was 2.68 ± 0.42 (Table 2).

**Table 2:** Knowledge about Medication Administration, Clinical Decision-Making, Nurse -physician Collaboration Satisfaction, and Perceived Barriers to Reporting Medication Administration Errors

Variables (number of items)	Mean ± SD	(*N* = 218)
		Actual range
Knowledge about medication administration (20)	0.65 ± 0.15	0.10~0.90
Clinical decision-making (24)	2.93 ± 0.11	2.63~3.42
Nurse -physician collaboration satisfaction (8)	4.01 ± 0.97	1.00~6.50
Perceived barriers to reporting medication administration error (16)	2.68 ± 0.42	1.31~3.75
Fear (5)	3.35 ± 0.65	1.60~5.00
Disagreement over medication error (5)	2.17 ± 0.53	1.00~4.00
Reporting effort (2)	2.86 ± 0.73	1.00~5.00
Administrative responses (4)	2.96 ± 0.59	1.00~5.00

### Correlations between individual self-efficacy/organizational bottom-up approach set and perceived barriers to reporting MAEs set

The assumptions of canonical correlation with regard to normality, linearity, homoscedasticity, and multicollinearity were met. The correlations between perceived barriers to reporting MAEs variables were .20–.52, indicating no within-set multicollinearity. The correlation between individual self-efficacy/organizational bottom-up variables and perceived barriers to reporting MAEs variables were |.16| -|.24|, which showed the absence of multicollinearity (Table 3).

**Table 3:** Correlations among Knowledge about Medication Administration, Clinical Decision-Making, Nurse -physician Collaboration Satisfaction, and Perceived Barriers to Reporting Medication Administration Errors

Variables				(*N* = 218)			
	Individual self-efficacy/Organizational bottom-up approach set			Perceived barriers to reporting medication administration errors			
	r*(p)*						
	1	2	3	4	5	6	7
Individual self-efficacy/ Organizational bottom-up approach							
1. Knowledge about medication administration	1.00						
2. Clinical decision-making	−0.01 (.868)	1.00					
3. Nurse-physician collaboration satisfaction	−0.11 (.114)	− 0.06 (.348)	1.00				
Perceived barriers to reporting medication administration errors							
4. Fear	0.08 (.234)	0.04 (.544)	−0.11 (.099)	1.00			
5. Disagreement over medication error	−0.24 (<.001)	0.16 (.019)	−0.04 (.548)	0.20 (.004)	1.00		
6. Reporting effort	0.04 (.581)	−0.04 (.532)	−0.16 (.019)	0.25 (<.001)	0.43 (<.001)	1.00	
7. Administrative responses	0.04 (.595)	0.11 (.102)	−0.22 (.001)	0.52 (<.001)	0.40 (<.001)	0.27 (<.001)	1.00

There were two pairs of significant canonical variates. The first canonical correlation was 0.37 and the second was 0.23. With all four canonical correlations included, Wilks’ lambda was 0.61(*F* = 4.19, *p* < .001); with the first canonical correlation removed, Wilks’ lambda was 0.89 (*F* = 2.88, *p* = .009). The first pair of canonical variates indicated that those with lower knowledge of medication administration (− 0.83), and inclined toward intuitive decision -making (0.41), and higher nurse -physician collaboration satisfaction (0.42), were associated with a higher perception of disagreement of over whether an error had occurred (0.64). The second canonical variate suggested that a combination of analytic decision –making (− 0.57) and higher nurse -physician collaboration satisfaction (0.84) was associated with lower perception of administration responses (− 0.94), disagreement over medication error (− 0.67), reporting effort (− 0.46), and fear (− 0.42) (Table 4).

**Table 4:** Canonical Correlations between Individual Self-efficacy/ Organizational Bottom-up Approach set and Perceived Barriers to Reporting Medication Administration Errors

Variables sets		(*N* = 218)
	Canonical variates	
	1st	2nd
Set 1: Individual self-efficacy/Organizational bottom-up approach		
Knowledge about medication administration	−.83	.12
Clinical decision-making	.41	−.57
Nurse -physician collaboration satisfaction	.42	.84
% Redundancy (r^2^)	4.58	1.83
Set 2: Perceived barriers to reporting medication administration errors		
Administrative responses	−.22	−.94
Disagreement over medication error	.64	−.67
Reporting effort	−.29	−.46
Fear	−.25	−.42
% Redundancy (r^2^)	2.02	2.24
*Canonical correlation*	0.37	0.23
*Significance test*: *F(p)*	3.84(<.001)	2.34(.031)
*Variance explained*	13.4	5.3

## Discussion

The study aimed to define the roles of individual/organizational factors in reducing perceived barriers to reporting MAEs using canonical correlations; therefore, the findings are discussed by focusing on the significant canonical variates among study variables.

We identified the perceived barriers to reporting MAEs according to the participants’ characteristics. Nurses with a master’s degree perceived lower barriers to reporting MAEs than the others, though it is hard to generalize the result because only three participants had a master’s degree. Education was a barrier for reporting MAEs in a previous research [34]. According to one study, more confident and autonomous and more experienced nurses might think that not all medication errors are severe enough to be reported [35]. However, because underreporting makes it impossible to accurately analyze the medication process behind errors or near misses, all errors, regardless of their severity, should be reported in order to ensure medication safety [2]. Emphasis on the benefits of reporting was provided to the nurses in continuing education or in their graduate coursework. To prove a stronger relationship, it is necessary to examine the relationship between educational status and barriers to MAE reporting after increasing the number of participants and to test the effectiveness of the educational program.

The highest perceived barrier to MAE reporting was fear, a result similar to a previous study [36]. Fear may come from Korean cultural attributes such as hierarchical culture and face-saving concern [36]. If nurses feel that they are working within a strongly hierarchical structure, they may be afraid of co-workers’ and unit managers’ potential reactions to MAE reporting, such as blame or punishment [6]. Face-saving is defined as an individual’s claimed sense of positive feelings in the context of social interactions [37]. Health professionals in conservative and collectivistic cultures are probably reluctant to report coworkers’ MAEs in order to maintain harmonious interrelationships [36]. This conservative culture and hierarchy may mean that trying to look for and report errors would be regarded as making their colleagues uncomfortable or even a betrayal. Therefore, chief executives in hospital organizations should try to build a non-punitive culture [38] and members of organizations need to think of reporting behavior as a way of improving patient-safety practice, not in terms of gaining or losing face.

There are two significant canonical variates in this data set, which means the variables in the individual self-efficacy/organizational bottom-up approach set are related to the variables in perceived barriers to MAE reporting. In the first significant pair of canonical variates, lower knowledge, intuitive decision-making, and good nurse-physician collaboration satisfaction were associated with a higher perception of disagreement over medication errors. Decision-making is based on how information is organized in memory and the weight given to the information, and insufficient knowledge might be related to more intuitive decision-making [15]. Intuitive decision-makers show rapid information processing, simultaneous cue use, and evaluation cue use, because this type of decision-making is based on immediate apprehension of relevant situations [39]. Since nurses use an intuitive approach in data collection and an analytic approach in data processing and the identification of problems [40], a mismatch between decision-making style and situation is present. Intuitive decision-makers cannot make decisions based on their insufficient error-related knowledge; therefore, nurses’ decision-making style may be transformed from intuitive to analytical. Supportive social and institutional contexts such as good collaboration satisfaction play an important role in decision-making [41]. In these conditions, when opportunities for discussion are provided, which may or may not be appropriate, intuitive decision-making would be fostered. To increase the precision of our understanding of their relationship, additional research needs to be conducted.

In the second pair, analytic decision-making and higher nurse-physician collaboration were associated with lower perceived barriers to error reporting. In clinical situations, smooth communication and cooperation not only enhance perceptions of being a team, lead to positive patient outcomes, and limit MAEs, but also promote informal error reporting among colleagues [6], and foster collaboration in decision-making [42]. In a previous study, when nurses were provided with potential error-driven situations, they would informally report errors as a means of clarifying and validating their opinion with their colleagues [6]. In addition, because formal error reporting is also regarded as a form of bottom-up communication [43], we suggest that staff-led communication and better teamwork must be established for better informal and formal error reporting. Analytical decision-making also needs to be carried out by healthcare professionals to reduce perception of barriers to reporting MAEs [15]. One strategy for supporting analytical decision-making may be the development of a clinical decision-making supporting system (CDSS) for error reporting. A CDSS is a computerized system that uses case-based reasoning rooted in treatment guidelines to assist clinicians in clinical decision-making [44]. It can improve medication safety by improving the process of care, such as by reducing medication errors and improving patient outcomes [45]. It is essential to use consistent terms [46, 47], such as those provided by a standardized taxonomy, when adopting a CDSS in error-reporting. It appears likely that a CDSS based on a medication-error taxonomy would facilitate analytic decision-making for error -reporting.

Better knowledge about medication administration and good nurse-physician collaboration satisfaction were the stronger factors in each significant pair of canonical variates for reducing perceived barriers to MAE reporting. According to a literature review, nurses’ use of clinical reasoning to maintain safe medication administration was inadequate and clinical reasoning required in-depth knowledge [48]. Based on this study, knowledge about medication administration will not only be used in clinical reasoning for safe medication, but also as a precursor of error reporting. However, it is noteworthy that perception of disagreement over medication errors was the lowest barrier, even though the level of knowledge in this study was not high. Cognitive dissonance between perceived and real knowledge level is a possible reason. According to metacognition theory, if learners can accurately perceive the status of their knowledge, they will tend to think of themselves as good learners and complete work more efficiently [49]. Therefore, increased metacognition for knowledge of medication administration is needed.

A top-down approach with leadership from senior personnel takes time to create better patient-safety [50] and needs to be converted into a bottom-up approach to foster teambuilding and an open, fair environment. Better collaboration has been linked specifically to safety-relevant performance [51] such as fewer hospital-acquired infections [52]. According to a recent study, nurses perceive physicians not as team members, but as independent healthcare workers [21]. In addition, nurses who perceive more respect perceive higher levels of nurse-physician collaboration [21]. When nurses work in an environment that supports and recognizes their professional role, they may feel respected, and therefore more confident participating in decision-making during the collaboration process [53] It is helpful if hospital administrators create management regulations for collaborative nurse-physician decision-making and to maximize nurses’ involvement in the decision-making process [21]. However, self-help efforts for respectful communication between healthcare providers, which may be the foundation for collaboration, are even more important. Therefore, to improve nurse-physician communication, communication tools such as SBAR and cross-training/interprofessional educational programs would be helpful [53].

## Limitations

Although this study provides meaningful results and findings, there are several limitations. A convenient sampling may suggest possible sampling bias; therefore, generalizability of the findings of this study to other settings is limited. Another important limitation is the relatively low reliability of medication administration knowledge, which means a low proportion of true variability to the total obtained variability. Therefore, instrument refinement and additional studies are necessary.

## Conclusions/implications for practice

Despite these limitations, this study showed the possible influence of an individual self-efficacy/organizational bottom-up approach on reducing perceived barriers to MAE reporting. The findings suggest three recommendations for the clinical and policy phases. In the clinical phase, medication administration education, including high-alert drug administration training for nurses, may reduce disagreement about whether errors have occurred. In addition, a bottom-up approach strategy such as improving collaboration with interactive communication may facilitate MAE reporting. In the policy phase, administrators need to make an effort to promote analytical decision-making for reporting MAEs. Hospital leaders who construct and implement evidence-based medication error reporting systems that support decision-making can reduce the perceived barriers to reporting MAEs. The present study sheds light on the broader view of reducing perceived barriers to reporting MAEs and suggests further research that examines the effectiveness of the experimental study to extend the strategy of enhancing MAE reporting.

## Data Availability

The datasets generated and/or analysed during the current study are not publicly available due but are available from the corresponding author on reasonable request.

## Abbreviations

ANOVA: Analysis of Variance
CDSS: Clinical Decision-making Supporting System
CVI: Content Validity Index
IRB: Institutional Review Board
KR: Kuder-Richardson
MAE: Medication Administration Error
RN: Registered Nurse
UK: United Kingdom
US: United States
